# Effect of Pelvic Floor Workout on Pelvic Floor Muscle Function Recovery of Postpartum Women: Protocol for a Randomized Controlled Trial

**DOI:** 10.3390/ijerph191711073

**Published:** 2022-09-04

**Authors:** Hongmei Zhu, Di Zhang, Lei Gao, Huixin Liu, Yonghui Di, Bing Xie, Wei Jiao, Xiuli Sun

**Affiliations:** 1 Department of Obstetrics and Gynecology, Peking University People’s Hospital, No. 11, Xi-Zhi-Men South Street, Xi Cheng District, Beijing 100044, China; 2 Department of Sports medicine and rehabilitation, Beijing Sports University, No.48, Xin Xi Road, Hai Dian District, Beijing 100084, China; 3 The Key Laboratory of Female Pelvic Floor Disorders, Beijing 100044, China

**Keywords:** pelvic floor muscle training, pelvic floor workout, pelvic floor muscle, pelvic floor dysfunction, postpartum, rehabilitation

## Abstract

Background: There is a risk of pelvic floor dysfunction (PFD) from baby delivery. Many clinical guidelines recommend pelvic floor muscle training (PFMT) as the conservative treatment for PFD because pelvic floor muscles (PFMs) play a crucial role in development of PFD. However, there is disagreement about the method and intensity of PFM training and the relevant measurements. To pilot the study in PFM training, we designed a Pelvic Floor Workout (PEFLOW) for women to train their pelvic through entire body exercises, and we planned a trial to evaluate its effectiveness through comparing the outcomes from a group of postpartum women who perform PELFLOW at home under professional guidance online with the control group. Methods/design: The randomized controlled trial was projected to be conducted from November 2021 to March 2023. A total of 260 postpartum women would be recruited from the obstetrics departments of the study hospital and women would be eligible for participation randomized into experimental or control groups (EG/CG) if their PFM strength are scaled by less than Modified Oxford grading Scale (MOS) to be less than grade 3. Women in EG would perform a 12-week PEFLOW online under the supervision and guidance of a physiotherapist, while women in CG would have no interventions. Assessments would be conducted at enrollment, post intervention (for EG) or 18th to 24th week postpartum (for CG), and 1 year postpartum. Assessment would be performed in terms of pelvic floor symptoms, including MOS, cough stress test, urinary leakage symptoms, pelvic organ prolapse quantitation (POP-Q), and vaginal relaxation, clinic examinations including Pelvic floor electrophysiological test, Pelvic floor ultrasound and Spine X-ray, overall body test including trunk endurance test, handgrip test, body composition test, and questionnaires including International Physical Activity Questionnaire Score-Short Form(IPAQ-SF), Pelvic Floor Distress Inventory Questionnaire-20 (PFDI-20), Pelvic Floor Impact Questionnaire-7 (PFIQ-7), the 6-item Female Sexual Function Index (FSFI-6), and the Pittsburgh Sleep Quality Index (PSQI). Primary analysis will be performed to test our main hypothesis that PEFLOW is effective with respect to strengthen PFM strength. Discussion: This trial will demonstrate that pelvic floor-care is accessible to most women and clinical practice on PFD may change relevantly should this study find that Online PEFLOW approach is effective to improve PFMs. Trial registration: ClinicalTrials.gov, NCT05218239.

## 1. Introduction/Background

Pelvic floor dysfunction (PFD) is a series of symptoms of dysfunction caused by injuries on pelvic floor muscle, nerve, ligament, and fascia injury. PFD has a serious negative impact on the common life of woman, who would experience different degrees of urinary incontinence, pelvic organ prolapse, chronic pelvic pain, sexual dysfunction, etc. Although not clinically emergent, PFD brings a heavy economic burden to the women who suffers from this problem. The latest updated data show that the prevalence of urinary incontinence in Chinese women reaches 30.9%, the incidence of symptomatic pelvic organ prolapse is up to 9.6% [1], and the prevalence of sexual dysfunction is about 29.7% [2]. Pelvic floor diseases are more prevalent among women who have multiple births.

Pregnancy and vaginal delivery have independently been proved to be the risk factors for the development of severe urinary incontinent (SUI) as they could obviously weaken the pelvic floor muscle (PFM) strength. However, it was hard to evaluate patients’ muscle strength objectively; therefore, it was hard to validate whether a measure could be effective to have the weakened pelvic floor muscles be recovered before Modified Oxford scale (MOS) for muscle strength was published [3]. MOS provides quantitative scales for muscle strength, and it is simple and easy to apply clinically. It is becoming widely accepted method for pelvic floor muscle strength assessment since it was published. Literately and clinically, MOS 4 and 5 is identified as having “good” or “strong” muscle contraction which represents a normal condition of the pelvic floor muscles; relevantly MOS ≤3 indicates a weak contraction or no contraction being felt [4]. Some studies suggested that pelvic floor muscle strength be correlated with the incidence of pelvic organ prolapse (POP). Thus, Pelvic Organ Prolapse Quantification (POP-Q) was developed for evaluation of the pelvic floor muscle [5]. Borello-France et al. [6] used POP-Q to evaluated women’s pelvic floor muscle in their studies and argued that women with stage II of prolapse were better than those with stage III or IV in elevating their pelvic floor, and also would be easier to get benefit from PFM training than those over stage III. Scientific evidence showed that 10–20% of patients who were not intervened for the clinically confirmed POP got progressed in term of POP-Q scoring in 2-year follow-up [7,8], so early prevention or treatment should have high priority. The incidence rate of POP is higher in middle aged and elderly women. PFM training is recommended to women postpartum for it reduces the incidence of PFD during 6–12 months postpartum [9] and should possibly reducing the incidence of diseases from muscle dysfunction related to the anatomical changes with aging [10]. A program of supervised PFM training was recommended as a first option for women with symptomatic POP-Q stage I or stage II [11]. MOS and POP-Q can be the objective indicators be used to validate the effectiveness of any measure in improving PFM.

PFM training is the most popular conservative intervention for PFD. Its role in PFM improvement is becoming more and more important along with the rapid development of rehabilitation medicine and it is more and more acceptable by women as their requirements for improvement of quality of life are getting stronger. There are many kinds of programs for post-delivery exercises that are commercially available for women. Formulating a rehabilitation program after childbirth is certainly an effective strategy for most women to benefit from the post-delivery recovery. However, PFM rehabilitation relied on the synchronous recovery of both type I and type II muscle fibers, and, as it is difficult to identify type I and type II of the muscle fibers accurately by nonprofessionals during pelvic floor muscle contraction, exercises are usually not properly programmed, and the effectiveness of such programs are not easy to gain. Formulating a science-based rehabilitation program that can trained type I and type II muscle fibers for their synchronous rehabilitation after childbirth is certainly an effective strategy for most women to benefit from the post-delivery recovery.

Many pelvic floor rehabilitation methods were explored in previous studies. In 1948, Kegel training was first proposed and adopted as the gold standard of pelvic floor muscle training (PFMT) [12]. PFMT refers to the conscious exercise via autonomic contraction of the pelvic floor muscles that are dominated by pubococcinate muscles. The American Urogynecologic Society evaluated PFMT as the wisest exercise choice for women and recommended it as the first-line treatment for PFD [13]. Kegel exercise has rarely been used alone until now. The human body is an organic whole, so PFMT should not be limited to the local pelvic floor. In 1980, abdominal hypopressive technique (AHT) [14] was invented. It emphasizes PFMT to be combined with activation of transverse abdominis muscle and diaphragmatic breathing [14]. Some studies found that training of the core muscles, including rectus abdominis, transversus abdominis, internal or external oblique, lumbar multifidus, and erector spinae muscles [15] for their stability, such as Yoga exercises, can strengthen PFM [16,17]. A French therapist, Philippe, proposed a concept of Global Postural Re-education (GPR) [18,19] and demonstrated that GPR was advantageous in improving respiratory muscle strength and reducing urinary incontinence since GPR was projected to correct postural misalignments by stretching the muscle chains [19]. In consideration that the spine is a continuous boney structure that is closely associated with pelvic dynamics [20], some investigators in recent years focused their research on restoring the balance of anatomic mechanical systems through adjustment of the posture. It was found that therapeutic exercise on posture adjustment (diaphragm and lumbar position) for patients with poor respiration and posture can improve the neuromuscular control on muscles of deep abdomen, diaphragm, and pelvic floor, and promote the stability of lumbar and pelvic floor. In addition, a significant correlation between overall posture and PFD was also demonstrated in several studies [20,21].

In 2020, the Canadian Society of Obstetricians and Gynecologists (SOGC) recommended that, to gain effectiveness, PFMT should be performed under supervision for at least 3 months [22]. Some previous studies [23,24,25,26] in alleviation of symptomatic ureter leakage demonstrated that physical exercise with PFMT was effective on the pelvic floor muscle.

In referring to the above published studies and concepts and based on the previous study [27] of our team, our team from Peking University Peoples Hospital (PKUPH) developed a Pelvic Floor Workout (also named as PKUPH-PEFLOW), which focuses on training to improve the strength, endurance, flexibility, stability, and flexibility of core muscles, including diaphragmatic, abdominal, pelvic floor, and lower back muscles. We also designed a randomized controlled clinical trial to demonstrate the effectiveness of PEFLOW. The trial was designed on hypothesis that (1) a 12-week Global Pelvic Floor Workout would improve the pelvic floor muscle strength of postpartum women; (2) the 12-week Global Pelvic Floor Workout would positively impact on the overall function of postpartum women, including pelvic floor function, body posture, pain, etc.; and (3) body posture, physical activity level, trunk muscle endurance and strength would have beneficial effects on maternal overall function.

## 2. Methods

### 2.1. Study Design

This is a randomized controlled trial to be conducted from November 2021 to March 2023. Participants are recruited from the obstetrics departments of Peking University People’s Hospital. The study was approved by Peking University Institutional Review Board (number: 2021PHB254-001) and registered in Clinical.Trials.gov (number: NCT05218239). 

### 2.2. Inclusion Criteria

Postpartum women will be eligible for enrollment if they are: (1) in 6th to 12th week postpartum; (2) without history of clinically confirmed systematic diseases, such as neurological conditions and cardiac insufficiency, currently not wearing cardiac pacemaker or receiving hormone therapy, and/or not been treated with radical surgery for pelvis and sling or surgery for prolapse before pregnancy, and with no pregnant complications; (3) willing to participate signing informed consent form; and (4) able to read without cognitive problem from poor education or diagnosed mental problem.

### 2.3. Exclusion Criteria

Participants will be excluded if they are: (1) confirmed by postpartum pelvic examination to be >grade 3 of pelvic floor muscle strength by Modified Oxford Scale (MOS); (2) ≥stage III for Pelvic Organ Prolapse Quantification (POP-Q); (3) symptomatically confirmed to have severe urinary incontinence (Women with more than one incontinent episode per day AND an important volume of urine loss [28] or urine leakage occurred in a quiet state were classified as having severe urinary incontinence); or (4) any limb dysfunction that unenabled women to take this training. A participant will be recorded as“ drop off from trial” if she becomes pregnant during the follow-ups.

### 2.4. Site Investigators and Roles

To conduct and evaluate the project, the study should assign the following persons to play roles: (1) a gynecologist (can be the chief investigator) who would conduct baseline examination and evaluation for eligible women, and another two gynecologists who would conduct examinations at the second and third follow-up visits, respectively, who was unaware of the data from the last follow-up. The examinations would include MOS, POP-Q, cough stress test, and vaginal relaxation.

(2) A research coordinator who is responsible for informed consent, study record and data management, contacting with and be accompanied with the participants during the examinations.

(3) A physiotherapist who would apply the designed intervention to participant in experimental group (EG).

(4) A physiotherapist assistant who would be responsible for correcting actions of EG during training and record Borg’s Rating of Perceived Exertion 6-20 (RPE) Scale after each session of training. (5) Clinic examiners who would be responsible for pelvic floor surface electromyography (sEMG), pelvic floor ultrasound examination, and Spine X-ray. 

### 2.5. Randomization and Allocation Concealment

Eligible women would be randomized into two groups, the experimental group (EG) and the control group (CG), at a ratio of 1:1. A randomization list would be created by a statistician from Peking University for each center before the start of the research. Randomization would be performed using SAS 9.4 (SAS Institute, Cary, NC, USA). The randomization lists would be concealed until a woman is confirmed eligible for participation. Then, the research coordinator in the site would assign the eligible participant to EG or CG. With such a randomization, the numbers of participants in EG and CG are expected to be statistically balanced. Participants and the physiotherapists engaging in the intervention are not blind to the randomized assignment because it is not feasible to do. However, to minimize the risk of assessor’s unblinding, participants are blind to other’s assignments and encouraged not to discuss their treatment with the independent assessor in the informed consent procedures and at the time of each assessment. 

### 2.6. Assessment Schedule

**Figure 1:**
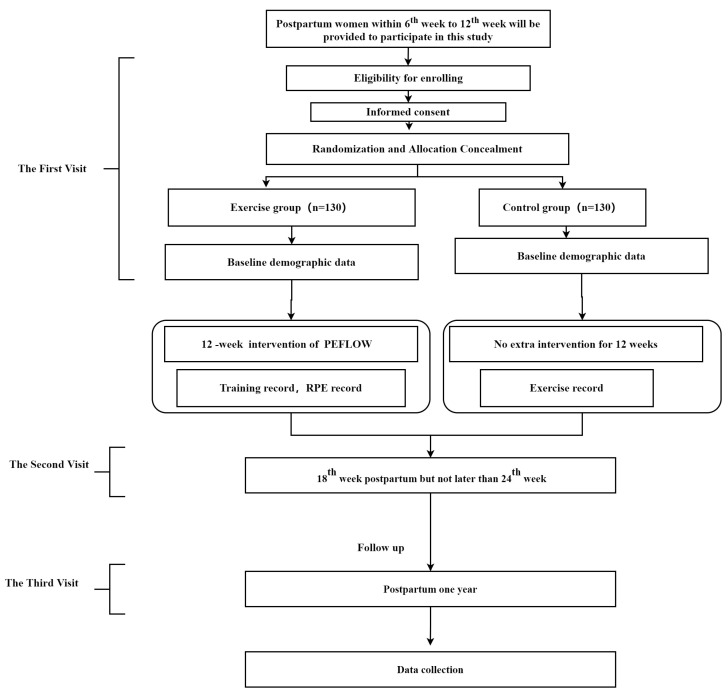
Flowchart of the trial.

Each participant would experience three assessments to go through the whole procedures in this study. The first assessment is for enrollment and would be given when women is confirmed eligible for participation. The second assessment would be conducted after 18th week postpartum but not later than 24th week. The final assessment would be performed in one year after delivery (Figure 1).

### 2.7. Interventions

All participants in EG and CG would be undergo postpartum pelvic floor evaluation according to standard comical procedures, which includes instructions for PFM strengthening with Kegel [12] and Knack [29] methods to improve voluntary contraction of the pelvic floor muscles, as well as daily living habits and posture education, such as reducing stool time, avoiding heavy lifting, etc. No extra intervention would be given to the participants in CG. 

In addition to the clinical standard pelvic floor evaluation, participants in EG would be provided with a Pelvic Floor Workout (PEFLOW). PEFLOW is a general program for physical training of the pelvic floor via entire body exercise. PEFLOW trains on coordination of breathing and muscle movement, maximum contraction of PFM, quick contraction of the PFM, and enduring contraction of PFM. The frequency of exercise following PEFLOW is 2 sessions a week for 12 weeks. It should be performed under the guidance of the professional physiotherapist for the whole process. It is designed for offline and online exercise. Due to COVID-19 pandemic prevention, PEFLOW in this study would be performed by the participants in EG at home under the online guidance of 2 physiotherapists. In order to keep the training effective, we would conduct the training in grouping [26] with each including less than 10 persons and ensure that no woman is over than 12 weeks postpartum at her first session of PEFLOW.

The Physiotherapist-online-guided PEFLOW (OG-PEFLOW) would be performed by participants in EG twice sessions a week for 12 weeks (Figure3). OG-PEFLOW would be provided via Zoom and in the form of live broadcasting. To monitor the performance of the participants, the participants would be requested to turn on the camera on their playing devices during the whole sessional procedures; however, they would be fully informed that no video or picture would be recorded or shot to protect their privacy. In each session of the OG-PEFLOW, a physiotherapist would perform exercise guidance, while another physiotherapist would monitor the performance of the participants and take notes. Details of the PEFLOW will be published in another manuscript. 

During the 12 weeks of intervention, participants in EG group would be requested to record their training load using RPE scale [30] every time after each training session. RPE quantitative evaluation of exercise load is calculated by “RPE score times x Exercise duration”. RPE scale indicates the exercise intensity in a specific time and the participants’ subjective psychological response to the stimulation from the exercise loads. In addition, Both EG and CG needs to record their training or exercise (if any) from the 6th week to 1 year postpartum. All of those records would be used for post-intervention assessment after 12-week intervention of EG.

The PEFLOW (Table 1 and Table 2) is a multimodal exercise program including moderate-intensity, aerobic, resistance, balance, and stretching exercise combined with PFMT, and the duration of each training session is about 60 min. Included in this program are ten sections, one for pelvic floor warm-up, eight for global exercises, and one for cooling down.

All participants in EG are informed to claim to the medical providers involved if any experiencing symptoms related to sport injury (e.g., knee pain, elbow pain, etc.). Any patients claiming to have such injury would be referred to the obstetric department or the physio therapist for treatment followed by an assessment for whether they should continue the training or not. An anticipated adverse event log would be prepared and sent to the principal investigator (PI) when such injury happens.

## 3. Data Collection and Management

Demographic data, including the age, height, weight, body mass index (BMI), prenatal maximum weight, physical activity during pre-pregnancy and pregnancy, and background data including the working position, smoking, alcohol consumption, urine leakage, asthma, constipation, family history of PFD, delivery history of pregnancy, weight gain during pregnancy, and fetal weight, would be collected at the enrollment visit of the study; while the following questionnaires would be filled by all participants at baseline and at all follow-up visits:

(1) The International Physical Activity Questionnaire Score-Short Form (IPAQ-SF), which were recommended as a cost-effective method to assess physical activity by nine questions recording the activity of four levels of intensities in short forms: vigorous-intensity, moderate-intensity, walking, and sitting [31];
(2) The Pelvic Floor Distress Inventory Questionnaire-20 (PFDI-20) and Pelvic Floor Impact Questionnaire-7 (PFIQ-7), which were designed to comprehensively evaluate to what extent the lower urinary tract, lower gastrointestinal tract, and pelvic organ prolapse symptoms affect the quality of life of women who suffer from PFD [32];
(3) The Female Sexual Function Index (FSFI-6) [33], a common tool that has been validated for clinical use for evaluating female sexual function in six terms [34,35];
(4) The Pittsburgh Sleep Quality Index (PSQI), which was developed in 1989 and accepted as the most common measure of sleep quality [36].

Baseline demographic data, IPAQ-SF, PFDI-20, PFIQ-7, FSFI-6, and PSQI were integrated into a WeChat mini program and can be filled by the Participants themselves by scanning a QR code on WeChat, a social App that is commonly used by the public in China. The body mass index (BMI) among the demographic data and all the scores or grades for all the questionnaires can be automatically come out by the configured formulas in the WeChat mini program. 

Following the questionnaires in each of the visits would be the physical examination including PFM strength evaluation by Modified Oxford Grading (MOS) [3], pelvic organ prolapse quantitation (POP-Q) stage [5], vaginal relaxtity, symptomatic SUI detection and grading, pelvic floor surface electromyography (sEMG) based on Glazer protocol [37], pelvic floor ultrasound examination, quantitative measurement of pelvic sagittal rotation degree by X-ray, and evaluation of the overall body composition and strength. 

Pelvic floor surface electromyography (sEMG) would be tested based on Glazer protocol. The participants would be taught to contract the pelvic floor muscle correctly and will have one practice before testing.

SUI is defined by SUI symptoms or a positive result of the cough stress test. Before the cough stress test, participants are suggested to keep fully bladder. Positive result of the test is defined as involuntarily urinary leakage when asked to cough in the lithotomy position.

Pelvic floor ultrasound indicators, which include LAM thickness, diameter of the levator hiatus, and levator hiatus area, were measured using the proprietary software 4D View via transperineal ultrasound(GE Kretz Medizintechnik), a reproducible and reliable measurement of vaginal support [38].

The quantitative measurement of pelvic sagittal rotation degree on Spine X-ray (Definium7000, General Electric Company, Boston, MD, USA) was analyzed which includes the pelvic tilt angle, the sacral slope angle, the difference of sacral slope and pelvic tilt, the ratio of the pelvic tilt angle to the sacral slope angle, the ratio of the pelvic tilt angle to the pelvic incidence angle and sacral-femoral distance.

The change of the overall body composition would be analyzed through body composition analyzers (Inbody 770), and overall strength which would be measured through hand grip strength test and trunk endurance test. Body composition analyzers would analyze data regarding waist hip ratio, basal metabolic rate, body fat rate, etc. Hand grip strength would be measured by JAMAR Plus+ grip gauge. The measurement accuracy is 0.1 kg. The participants should not have visible hand defects. The grip gauge should be adjusted before measurement. When the participants hold the grip gauge, the second knuckle of the index finger should be at 90 degrees. At the same time, mark the best position of each hand on the grip meter. Before measurement, the participants should remove wrist jewelry to avoid injury. The left and right hand are measured alternately three times. Trunk endurance would be tested using the Plank test, Side bend test, and Biering–Sorensen Test [39]. All the participants were encouraged to keep the positions as long as possible. Time as recorded in seconds with a maximal length of 90 s.

## 4. Outcomes of the Study

The primary outcome will be the comparison of participants’ pelvic floor muscle strengths using MOS scale at the enrollment baseline and post intervention. The secondary outcomes will be: (1) the occurrence of SUI, (2) the change of the Pelvic floor ultrasound indicators, (3) change of the Pelvic Organ Prolapse Quantitation measured by callipers, (4) change of the pelvic sagittal rotation degree, and (5) the change of the overall body composition and strength. The list of data collection instruments and time of data collection is presented in Table 2 and Table 3.

The other secondary outcome measures involved in the Table 2 will be calculated and discussed separately.

### 4.1. Sample Analysis

Sample size was calculated according to the improvement rate of MOS grade after 12-week training using PASS 2019 software. According to the preliminary experiment, the improvement rate of MOS in the CG was 20% and that of women in EG was 60% at 6 months postpartum. Group sample sizes of 106 in each group achieve 90.248% power to detect a difference between the group proportions of 0.4. The CG proportion is 0.2. The EG proportion is assumed to be 0.4 under the null hypothesis and 0.6 under the alternative hypothesis. The test statistic used is the one-sided Z test (unpooled). However, in considering 20% of the possible dropout, a total of 260 women will be randomized.

### 4.2. Statistical Analysis

Data will be analyzed using SPSS version 26.0 (SPSS, Inc., Chicago, IL, USA) and the statistically significant difference will be identified if *p* < 0.05. Descriptive statistics including numbers and proportions, means and standard deviations, or medians (P25, P75) will be used. Group differences in the primary and secondary outcomes will mainly use chi-square and student’s t tests. For primary outcome, we will count the rate of pelvic muscle strength reaching grade 4 at 6 months postpartum between EG and CG by chi-square test or Fisher’s exact test as appropriate. Analysis of the primary and secondary outcomes will be based on an intention-to-treat basis in consideration of the drop-offs from the follow-ups and be conducted by the statistician at Peking University Clinical Research Institute. For comparison between groups of quantitative data, the analysis of variance or Wilcoxon rank sum test will be used according to the data distribution; chi square test or exact probability method is used for counting data (if chi square test is not applicable), Wilcoxon rank sum test or CMH test is used for grade data, and logistic regression analysis is used for correlation analysis. Repeated ANOVA will be used to assess within-patient correlation. and multivariable linear regression models if potential confounders were imbalanced.

## 5. Discussion

Pelvic floor rehabilitation is becoming more and more important due to the rapid development of rehabilitation medicine and the improvement of the life quality of demanded by women. PFMT is one of the widely adopted exercise therapies in the clinic for female pelvic floor rehabilitation.

Although medical societies realize that rehabilitative exercises to be the currently demonstrated effective way to improve PFM, consensus on the standard for PFMT is still lack. Many issues are remaining; questions, such as “what is the right to exercise correctly?” and “what is the proper frequency and duration for PFM training?”, and reports on the efficacy of PFMT are much confusing. Due to the positional particularity and abstractness of pelvic floor muscles, it is difficult for a non-medical backgrounded woman to exercise pelvic floor muscle fiber I and fiber II directly, accurately, and/or effectively. PFMT has not been skilled for public populization, although many investigators have efforted exploring more convenient and effective pelvic floor rehabilitation methods.

In referring to comprehensive literature related to our previous research [20], we developed 12-week PEFLOW, with emphases on training of the entire muscle system. 

PEFLOW is composed of 10 sections as shown in Table 1. The warm-up section was setup to have the participants percept the pelvic floor contractions in different body positions via self-feeling to the pelvic floor movements. In this section, participants are instructed to practice quick contraction (1”) and relaxation (2”) for perception of type II muscle fiber (fast-twist fiber) and hold-on contraction (8-10”) and relaxation (8-10”) for perception of type I muscle fibers (slow-twist fiber). The training of multiple repetitions for fast-twist fibers is also a way of targeting slow-twist fiber [41]. Section 1 was integrated in the program to train the pelvic contractions corelating to the movements of standing, heel lifting, single feet standing, and walking. According to the literature, feet play roles in control and adjustment of postures, and exercises corelated with the movement of feet can improve body balance and simulate feedback perception [42]. Section 2 was setup to improve the flexibility and mobility of the lumbar vertebrae and pelvic, so as to make them adapt the changes in woman’s body postures that is the unavoidable outcome of pregnancy and delivery due to the changes in lumbar vertebrae bulge, pelvic width and tilt, and the relevant muscle tension [43]. With Section 3, we tend to train the movements of PFM with correlating to breaths. It was programed based on a rational from multiple studies that exhaling maximizes PFM tension and strength [14,44]. Sections 4–8 were programed to train joint contractions of the core muscles, surrounding muscles and PFMs as what more and more scientific evidence demonstrated that PFM contraction can be optimized by joint contractions of the core muscles such as abdominal, lumbar dorsal, and diaphragm muscles [40], and surrounding muscles such as hip abductors [45,46].

Optimal and long-term therapeutic effects are anticipated. We made PEFLOW be performed at home due to the meeting restrictions in COVID-19 pandemic. However, we anticipate that home exercise with the facilitation of online guidance and encouragement will increase the participation rate and the concordance of the women in taking correct exercise. The requirement for participants to report the RPE score after each training session is not only an encouragement to the participants, but a guarantee of the appropriate training intensity being kept training effectively and safely [47].

The possible limitation of this trial might be that we lack more precise measurements for physical activity level, such as wrist or waist accelerometers to monitor heart rate during daily physical activities and trainings.

## 6. Conclusions

In conclusion, this study is expects that the 12-week online PEFLOW is a cost-effective training for the recovery of Pelvic floor muscle function, which is suitable for future clinic trails and also for clinical promotions.

## Figures and Tables

**Table 1:** The 12-week Pelvic Floor Workout and the sectioning.

	Week	8-Scetion Program	Week	8-Scetion Program
**Warm-ups**	Pelvic floor perception, try to feel the contraction of pelvic floor muscles at different positions, including standing, lying and sitting position.			
**Main** **Progressive Exercises**	1	Standing squeezing Sitting on Swiss ball: pelvic dancing Lying: new respiratory exercise Lying: hip curling Lying: dynamic hand-knee resistance Side lying: knee bending side bridge Lying: alternating knee extension Four-foot kneeling: alternating knee extension	7	Walking squeezing Sitting on Swiss ball: pelvic with up-limb dancing Lying: new respiratory exercise with balloon Lying: hip isometric curling with balloon Lying with neck bending: leg clip ball+ isometric hand-ball resistance Side lying: knee extension isometric side bridge Lying: four-foot dynamic extension with elastic belt Four-foot kneeling: four-foot dynamic extension with elastic belt
	2	Standing squeezing Sitting on Swiss ball: pelvic dancing Lying: new respiratory exercise Lying: hip curling Lying: dynamic hand -knee resistance Side lying: knee bending side bridge Lying: four-foot dynamic extension Four-foot kneeling: four-foot dynamic extension	8	Walking squeezing Sitting on Swiss ball: pelvic with up-limb dancing Lying: new respiratory exercise with balloon Lying: Hip isometric curling with balloon Lying with neck bending: leg clip ball+ isometric hand-ball resistance Side lying: knee extension isometric side bridge Lying: four-foot dynamic extension with elastic belt Four-foot kneeling: four-foot isometric extension with elastic belt
	3	Calf raising squeezing Sitting on Swiss ball: pelvic dancing Lying: new respiratory exercise Lying: Hip curling Lying with neck bending: dynamic hand -knee resistance Side lying: knee bending “clam” side bridge Lying: four-foot dynamic extension Four-foot kneeling: four-foot dynamic extension	9	Walking squeezing +Arm large swing Sitting on Swiss ball: pelvic with up-limb dancing using elastic belt Lying: new respiratory exercise with balloon Lying: hip isometric curling with balloon Lying: neck bending +Leg Clip Ball+ Side ball shot Side lying: knee extension side bridge with arm cross swing Lying: four-foot dynamic extension with elastic belt Four-foot kneeling: four-foot isometric extension with elastic belt
	4	Calf raising squeezing Sitting on Swiss ball: pelvic dancing Lying: new respiratory exercise Lying: hip curling with balloon Lying with neck bending: dynamic hand -knee resistance Side lying: knee bending clam side bridge Lying: four-foot isometric extension Four-foot kneeling: four-foot isometric extension	10	Walking squeezing +Arm large swing Sitting on Swiss ball: pelvic with up-limb dancing using elastic belt Lying: new respiratory exercise with balloon Lying: hip dynamic curling with Swiss ball and balloon Lying with neck bending: Leg Clip Ball+ Side ball shot Side lying: knee extension side bridge with arm cross swing Side Lying: Four-foot isometric extension with elastic belt Four-foot kneeling: four-foot isometric extension with elastic belt on unstable support
	5	Single leg squeezing Sitting on Swiss ball: pelvic and up-limb dancing Lying: new respiratory exercise Lying: hip curling with balloon Lying with neck bending: isometric hand -knee resistance Side lying: knee extension dynamic side bridge Lying: four-foot isometric extension Four-foot Kneeling: four-foot isometric extension	11	Walking squeezing with arm small swing Sitting on Swiss ball: pelvic with up-limb dancing using elastic belt Lying: new respiratory exercise with balloon Lying: hip dynamic curling with Swiss ball and balloon Lying with neck bending: leg clip ball+ side ball shot Side lying: four-foot extension side bridge Lying: four-foot isometric extension with elastic belt Four-foot kneeling: four-foot isometric extension with elastic belt on unstable support
	6	Single leg squeezing Sitting on Swiss ball: pelvic and up-limb dancing Lying: new respiratory exercise Lying: hip curling with balloon Lying with neck bending: isometric hand -knee resistance Side Lying: knee extension dynamic side bridge Lying: four-foot isometric extension Four-foot Kneeling: four-foot dynamic extension with elastic belt	12	Walking squeezing with Arm small swing Sitting on Swiss ball: pelvic with up-limb dancing using elastic belt Lying: new respiratory exercise with balloon Lying: hip dynamic curling with Swiss ball and balloon Lying with neck bending: leg clip ball+ side ball shot Side lying: four-foot extension side bridge with upper leg abducted (if not, the same as week 11th) Lying: four-foot isometric extension with elastic belt Four-foot kneeling: four-foot isometric extension with elastic belt on unstable support
**Cooling Down**	Dynamic stretching, including upper and lower limbs, waist, back, and abdomen stretching and pelvic floor muscle relaxation with mini foam axis.			

**Table 2:** Data collection and follow-up instruments.

		EG	CG	EG	CG	EG	CG
Baseline screen, Informed consent, Randomization		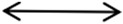					
The follow-up visits		The First		The Second		The Third	
Demographic data collection		◯	◯				
The MOS		●	●	●	●	●	●
POP-Q stage		●	●	●	●	●	●
Cough stress test		●	●	●	●	●	●
Vaginal relaxity		●	●	●	●	●	●
Pelvic floor electrophysiological test		◎	◎	◎	◎	◎	◎
Pelvic floor ultrasound		◎	◎	◎	◎	◎	◎
Spine X-ray		◎	◎	◎	◎	◎	◎
Body Composition		◎	◎	◎	◎	◎	◎
Hand grip test		◎	◎	◎	◎	◎	◎
Trunk endurance test		◎	◎	◎	◎	◎	◎
Questionnaires	PFDI-20	◯	◯	◯	◯	◯	◯
	PFIQ-7	◯	◯	◯	◯	◯	◯
	FSFI-6	◯	◯	◯	◯	◯	◯
	IPAQ-SF	◯	◯	◯	◯	◯	◯
	PSQI	◯	◯	◯	◯	◯	◯
Training record				✷	✷(if any)		
RPE				✷			
Adverse Event				☐	☐		

●: Data would be collected by pelvic floor professionals via vaginal palpation. ◎: Data would be collected by experienced General specialists as the examiners. ◯: data would be collected by requesting all participants to give answers through scanning a QR code. ✷: Recording by all the participants in EG only per their training record and RPE score given by themselves.: Optional items. Abbreviations: MOS: The modified Oxford Scale [3], which needs to be performed by inserting two fingers into the vagina for approximately 4 cm to feel the FPM contraction and give scale ranging from 0 (no contraction) to 5 (strong contraction).; POP-Q [5]: pelvic organ prolapse quantitation; PFDI-20: The Pelvic Floor Distress Inventory Questionnaire-20; PFIQ-7: Pelvic Floor Impact Questionnaire-7; FSFI-6: The 6-item Female Sexual Function Index; IPAQ-SF; International Physical Activity Questionnaire Score-Short Form; RPE: Ratings of Perceived Exertion.

**Table 3:** Outline of Soring for general pelvic floor function.

Testing Items	Indicators	Compute Value	Score Value
POP-Q	Stage 0	0	
	Stage I	1	
	Stage II	2	
	Stage III	3	
	Stage IV	4	
Cough stress test	Yes	1	
	No	0	
Vaginal relaxation	≥4 fingers	4	
	>3 fingers, and <4 fingers	3	
	=3 fingers	2	
	>2 fingers, and <3 fingers	1	
	≤2 fingers	0	
Pelvic floor electrophysiological test [40]	<20 cm^2^Hg	1	
	≥20 cm^2^Hg	0	
			* Total Score ^1^
PFDI-20	-	Comprehensive score	
PFIQ-7	-	Comprehensive score	
FSFI-6	-	Comprehensive score	
			* Total Score ^2^

* The total score ^1^ means the sum of all above score values which represents objective indicators for pelvic floor function. The lower the total score, the better the pelvic floor function; the total score ^2^ means the sum of the above three comprehensive score which represents women’s subjective evaluation by themselves for pelvic floor.

## Data Availability

Not applicable.
